# Thematic Integration Impairments in Primary Progressive Aphasia: Evidence From Eye-Tracking

**DOI:** 10.3389/fnhum.2020.587594

**Published:** 2021-01-06

**Authors:** Matthew Walenski, Jennifer E. Mack, M. Marsel Mesulam, Cynthia K. Thompson

**Affiliations:** ^1^Department of Communication Sciences and Disorders, Northwestern University, Evanston, IL, United States; ^2^Mesulam Center for Cognitive Neurology and Alzheimer’s Disease, Northwestern University, Chicago, IL, United States; ^3^Department of Neurology, Northwestern University, Chicago, IL, United States

**Keywords:** primary progressive aphasia, agrammatism, sentence comprehension, syntax, eye-tracking, visual world

## Abstract

Primary progressive aphasia (PPA) is a degenerative disease affecting language while leaving other cognitive facilities relatively unscathed. The agrammatic subtype of PPA (PPA-G) is characterized by agrammatic language production with impaired comprehension of noncanonical filler-gap syntactic structures, such as object-relatives [e.g., *The sandwich that the girl ate (gap) was tasty*], in which the filler (*the sandwich*) is displaced from the object position within the relative clause to a position preceding both the verb and the agent (*the girl*) and is replaced by a gap linked with the filler. One hypothesis suggests that the observed deficits of these structures reflect impaired thematic integration, including impaired prediction of the thematic role of the filler and impaired thematic integration at the gap, but spared structure building (i.e., creation of the gap). In the current study, we examined the on-line comprehension of object-relative and subject-relative clauses in healthy controls and individuals with agrammatic and logopenic PPA using eye-tracking. Eye-movement patterns in canonical subject-relative clause structures were essentially spared in both PPA groups. In contrast, eye-movement patterns in noncanonical object-relative clauses revealed delayed thematic prediction in both agrammatic and logopenic PPA, on-time structure building (i.e., gap-filling) in both groups, and abnormal thematic integration in agrammatic, but not logopenic, PPA. We argue that these results are consistent with the hypothesis that agrammatic comprehension deficits reflect impaired thematic integration.

## Introduction

Primary progressive aphasia (PPA) is a degenerative disease affecting language while leaving other cognitive facilities relatively unscathed (Mesulam et al., 2012). Three distinct subtypes of the disorder have been identified with different disease etiology and progression, as well as different profiles of spared and impaired language function. The agrammatic subtype of PPA (PPA-G) is characterized by the production of ungrammatical sentences and impaired comprehension of syntactically complex sentences, with spared single-word comprehension. In contrast, the logopenic and semantic subtypes (PPA-L, PPA-S, respectively) do not show grammatical impairments in production or comprehension. Rather PPA-L is characterized by impaired naming and word-finding with spared single-word comprehension, and PPA-S is characterized by impaired naming and single-word comprehension but spared speech production (Mesulam et al., 2009; Gorno-Tempini et al., 2011).

In this paper, we focus on the on-line mechanisms underlying the comprehension of complex noncanonical sentences in people with agrammatic PPA. Noncanonical sentences subvert the dominant *agent-verb-theme* order in English, such that the theme precedes the verb in these structures. The *agent* (the do-er of the verb’s action) and *theme* (the participant affected by the verb) refers to thematic roles that specify the semantic relation between the verb and its arguments. For example, in an object-relative clause structure [e.g., *The sandwich that the girl ate (gap) was tasty*], the theme (*the sandwich*) has been displaced from the object position following the verb [at the (gap) site] to a position preceding the verb (*ate*) and the agent (*the girl*). This displacement creates a filler-gap dependency, in which the displaced element (*the sandwich*) is the filler for the gap. In a subject-relative clause [*The girl that (gap) ate the sandwich ran away*], the agent (*the girl*) has been extracted from the subject position of the relative clause [i.e., the *(gap)* position], resulting in a preserved canonical *agent-verb-theme* order.

In sentences with noncanonical word order, linking the filler to the gap is essential for correct assignment of thematic roles, which in turn, is essential for comprehending *who did what to whom* within a sentence. While this process depends on syntax, many sentences can be understood even in the absence of syntactic processing. Thus in sentences with “non-reversible” relative clauses such as *The ball that the boy is kicking is round*, the thematic relations between the verb (*kicking*) and its arguments can be puzzled out based on real-world knowledge. In contrast, thematic relations in “reversible” relative clauses, where either participant could plausibly be linked to either thematic role, are difficult to understand without syntax, as real-world knowledge does not favor one interpretation over another. For example, *the eagle* in the sentence *The eagle that was chased by the hawk was faster* could plausibly be the agent or the theme of the relative clause verb (*chased*), likewise for *the hawk*. However, the syntax of this particular sentence indicates that *the eagle* (the subject) must be the theme and *the hawk* must be the agent. Without syntax, misunderstandings of such sentences would be common.

Sentences with reversible clauses, therefore, serve as a useful indicator of syntactic comprehension deficits. Indeed, deficient comprehension of reversible noncanonical structures is often found in agrammatic aphasia after a stroke (Caramazza and Zurif, 1976; Grodzinsky, 1986; Love et al., 2008; Thompson et al., 2013). In agrammatic PPA as well, the comprehension of reversible noncanonical constructions is consistently reported to be impaired, across a variety of experimental tasks, including sentence-picture matching (Wilson et al., 2010; Charles et al., 2013; Thompson et al., 2013; but see Zimmerer et al., 2014), sentence-picture verification (Kinno et al., 2017), and visual sentence decision (deciding if the agent is male or female; Cooke et al., 2003).

However, the results from these studies have all been based on off-line methods, in which the measurement occurs after the end of the sentence. Such off-line methods are temporally insensitive and allow for strategic, metalinguistic processing, as well as opportunities for self-correction and reflection, and so do not necessarily reflect the same (or even similar) processes that underlie automatic syntactic processing as a sentence unfolds over time (Swinney and Osterhout, 1990). While prior results may therefore speak to impaired sentence comprehension in agrammatic PPA, they do not reveal when or how it may have gone wrong.

On-line methods such as eye-tracking or event-related potential techniques are capable of measuring automatic sentence comprehension in real-time but have not been previously brought to bear on the comprehension of noncanonical structures in agrammatic PPA. On-line methods have been used to investigate language processing in agrammatic stroke aphasia, and given the similar profiles of off-line performance in the two disorders, theories of agrammatism developed in the stroke aphasia literature may well provide insight into the nature of on-line sentence comprehension deficits in agrammatic PPA.

There are many different perspectives on these comprehension deficits in aphasia (for review, see Kolk and Weijts, 1996; Dickey and Thompson, 2004; Patil et al., 2016). On one broad view, comprehension deficits in aphasia reflect a breakdown in grammatical knowledge. One prominent view from this perspective, the trace deletion hypothesis, posits that impaired comprehension of filler-gap structures reflects an inability to create gaps. Without the gap, the filler cannot be assigned a thematic role, and so comprehenders rely on heuristic mechanisms to interpret the role of the filler (Grodzinsky, 1986, 1995, 2000). However, tests of this hypothesis have been made almost exclusively with off-line measures such as sentence-picture matching, that do not measure how the sentence was processed before the response (Grodzinsky, 2000).

Alternative hypotheses focus on aspects of real-time processing during the comprehension of a sentence. One set of hypotheses suggest that the timing of key processes is disrupted in agrammatic aphasia. On these views, grammatical knowledge is posited to remain intact, but processing is slowed to the extent that the comprehension of filler-gap constructions is impaired. According to the *slow-syntax hypothesis*, the brain damage that results in agrammatic aphasia leads to a reduction in processing resources, such that syntactic structure building is slowed during real-time sentence comprehension (Burkhardt et al., 2003; Avrutin, 2006). To explain why the delayed but otherwise intact structure building does not ultimately succeed given enough time, on this view the slowed syntactic processing leads to interference from other processes that lead comprehension astray. On-line evidence from a cross-modal interference task suggests delayed processing of a filler at a gap during comprehension of noncanonical filler-gap constructions in agrammatic aphasia (Burkhardt et al., 2008).

According to the *lexical slow-rise hypothesis*, syntactic processing is neither impaired nor slowed in agrammatism. Rather, evidence from lexical priming studies suggests that lexical activation peaks later than normal in agrammatic aphasia (Swinney et al., 1996). This delayed lexical activation, therefore, feeds rapid automatic syntactic processing too slowly during normal-rate speech (roughly four to six syllables per second), leading to breakdowns in comprehension. Studies of sentence comprehension using cross-modal lexical priming report delayed priming effects both for the initial activation of a word and for the reactivation of the filler at a gap (Love et al., 2008; Ferrill et al., 2012). Also consistent with this view, presenting auditory sentences with a slowed input rate (less than four syllables per second) results in on-time re-activation of the filler at the gap and improved off-line comprehension in agrammatic listeners (Love et al., 2008).

On another view, difficulty comprehending complex syntactic structures in agrammatic aphasia reflects deficient thematic integration of verb arguments into the syntax (Thompson and Choy, 2009). This results, first, from failure to predictively assign the agent argument role to the first noun encountered (i.e., the filler) in noncanonical sentences, as do unimpaired listeners (Mack and Thompson, 2017) and, second, to re-assign the theme role to that argument when the gap is encountered. The results of several studies using eye-tracking paradigms with stroke aphasic agrammatic participants show eye movement patterns consistent with both thematic prediction and integration impairments (Meyer et al., 2012; Mack et al., 2013, 2016; Hanne et al., 2015). In contrast, the processes underlying the creation of the gaps in the syntax and reactivation of the filler at the gap position have been found to proceed normally (Dickey et al., 2007; Dickey and Thompson, 2009; Thompson and Choy, 2009).

In sum, while these hypotheses consistently predict off-line comprehension deficits in agrammatic aphasia for non-canonical structures, the time-course and nature of the predicted on-line deficits vary. Examining these predictions in agrammatic PPA therefore requires an on-line method with a fine-grained temporal resolution. Here, we use a visual world eye-tracking paradigm with reversible object-relative and subject-relative sentences presented auditorily at a normal speech rate. The paradigm and materials were adapted from those previously used in a study of agrammatic stroke aphasia, which reported on-time gap filling in sentences with object-relative clauses (Dickey et al., 2007; Dickey and Thompson, 2009).

According to the trace-deletion hypothesis, no evidence of reactivation of the filler at the gap site should be seen. According to the slow-syntax and lexical slow-rise hypotheses, gap-filling in sentences presented at a typical rate of speech should be qualitatively normal but delayed. According to the thematic deficit hypothesis, reactivation at the gap location in a noncanonical structure should be on-time in agrammatic PPA, but thematic role processes should be abnormal, both for thematic prediction (before the gap) and for thematic integration (at or downstream from the gap).

Finally, we include PPA-L as a comparison group. Offline comprehension deficits have been observed in PPA-L for noncanonical sentences, though such deficits may reflect verbal working memory deficits rather than grammatical deficits* per se*, and are not typically as severe as in PPA-G (Thompson and Mack, 2014). We are not aware of any on-line studies of noncanonical sentence comprehension in PPA-L.

## Materials and Methods

### Participants

Twenty individuals diagnosed with the agrammatic (*n* = 10) or logopenic (*n* = 10) variant of PPA and 15 healthy controls participated in the experiment (Table 1). All participants were monolingual native English speakers and were right-handed, based on the Edinburgh handedness questionnaire (Oldfield, 1971). All participants had normal or corrected-to-normal vision and hearing and reported no prior history of psychiatric illness or neurological disease. Also, we included only participants with PPA that passed a neurological exam that screened for abnormalities in vision and eye movement control, which are often associated with other neurodegenerative diseases, such as progressive supranuclear palsy (Leigh and Zee, 2015). Such screening appears to be sufficient to ensure that eye movement control is at least grossly normal in participants with PPA (Mack et al., 2019). The study was approved by the Institutional Review Board (IRB) at Northwestern University and all participants provided informed consent.

**Table 1:** Demographic, neuropsychological, and language measures of study participants.

Measure	Unimpaired adults	PPA: agrammatic	PPA: logopenic
	*N* = 15	*N* = 10	*N* = 10
Age (years)	65.5 (8.6)	61.4 (6.3)	65.8 (4.4)
Education (years)	15.6 (2.4)	16.1 (2.0)	17.4 (1.3)*
Sex	9M/6F	6M/4F	7M/3F
Symptom duration (months)	–	37.2 (21.6)	44.5 (30.1)
WAB-R aphasia quotient (100)	–	83.8 (8.8)	89.4 (7.9)
Clinical dementia rating (CDR)	–	1.15 (1.0)	0.65 (0.5)
MMSE (30)	29.3 (0.8)	24.4 (3.6)*	26.7 (2.4)*
Digit span forward (WMS-III)	7.9 (0.7)	5.1 (1.4)*	4.8 (1.5)*
Digit span backward (WMS-III)	5.8 (1.3)	3.3 (0.8)*	3.3 (0.8)*
WAB repetition subset (66)	65.3 (1.1)	47.8 (12.8)*	50.1 (9.8)*
**Language comprehension**			
PPVT (36)	35.5 (0.5)	33.0 (2.8)	34.1 (2.7)
PPT pictures (52)	51.3 (0.8)	48.6 (3.3)*	50.1 (1.3)*
NAVS verb comprehension test (%)	–	100 (0)	100 (0)
Canonical sentences: NAVS SCT (%)	–	93.3 (8.3)	98.7 (4.2)
Noncanonical sentences: NAVS SCT (%)	–	91.3 (9.9)	95.3 (7.1)
**Language production**			
NNB noun naming (%)	–	93.8% (9.3)	94.4% (13.4)
NNB verb naming (%)	–	88.1% (8.6)	93.1% (11.9)
Canonical sentences: NAVS SPPT (%)	–	86.7 (27.2)	96.7 (6.5)
Canonical sentences: NAT (%)	100 (0.0)	90.7 (9.0)*	96.0 (4.7)*
Noncanonical sentences: NAVS SPPT (%)	–	62.0 (30.3)	80.0 (15.7)
Noncanonical sentences: NAT (%)	99.3 (2.1)	46.7 (15.7)*^,L^	80.7 (13.1)*
Cinderella: words per minute	134.7 (16.3)	69.4 (19.0)*	81.6 (25.8)*
Cinderella: grammatical sentences (%)	97.1 (4.3)	70.1 (23.9)*	82.2 (8.0)*
Cinderella: MLU	11.3 (3.8)	8.1 (1.6)	8.9 (1.9)

Note: WAB-R, Western Aphasia Battery-Revised; CDR, Clinical Dementia Rating; MMSE, Mini-Mental State Examination; WMS-III, Wechsler Memory Scales, Fourth Edition; PPVT, Peabody Picture Vocabulary Test; PPT, Pyramids and Palm Trees Test; NAVS, Northwestern Assessment of Verbs and Sentences; SCT, Sentence Comprehension Test; SPPT, Sentence Production Priming Test; NNB, Northwestern Naming Battery; NAT, Northwestern Anagram Test; MLU, Mean Length of Utterance (words); *significantly different than unimpaired adults (*p* < 0.05, uncorrected); ^L^significantly lower than PPA-L (*p* < 0.05, uncorrected); –, not applicable or not administered. Neuropsychological and language measures were available for only 10 of the unimpaired adults.

The participants with PPA were diagnosed and classified into subtypes following the 2011 consensus criteria (Gorno-Tempini et al., 2011; Mesulam et al., 2012). All of the agrammatic participants had agrammatic language production; none were diagnosed as the agrammatic subtype based solely on speech apraxia. All participants completed a battery of standard language and cognitive assessments (Table 1). Unequal variance *t*-tests (two-tailed) were used to compare the groups pairwise on each measure (Zimmerman, 2004; Ruxton, 2006), except that *t*-tests were not conducted against the control group for tasks that the controls did not complete.

The participant groups did not differ significantly concerning age. The PPA-L group had more years of education than the unimpaired participants (*p* < 0.05), but there were no other significant group differences in education. The PPA groups did not differ concerning symptom duration, aphasia severity [Aphasia Quotients from the Western Aphasia Battery-Revised (WAB-R) Kertesz, 2006], or scores on the Clinical Dementia Rating scale (CDR; Morris, 1993), which indicated mild (if any) non-verbal cognitive impairment for both groups. Mini Mental-State Examination (MMSE; Folstein et al., 1975) scores were lower in both PPA groups compared to controls (*ps* < 0.05), likely reflecting impaired language (Osher et al., 2008). Working memory deficits, as measured by Digit Span Forward and Backward tests on the Wechsler Memory Scale-III (Wechsler, 1997) were evident both in the PPA-L and PPA-G groups relative to controls (*ps* < 0.05), but the two PPA groups did not differ from each other. Repetition of phrases and sentences (measured using a subset of items from the Repetition subtest of the WAB-R) was impaired relative to controls in both PPA participant groups (*ps* < 0.05), who did not differ from each other.

Tests of single-word comprehension and production showed that the two groups performed similarly for noun (object) comprehension [Peabody Picture Vocabulary Test (PPVT; items 157–192); Dunn and Dunn, 2006], verb (action) comprehension [Verb Comprehension Test from the Northwestern Assessment of Verbs and Sentences (NAVS; Thompson, 2011)], and object and action naming [Northwestern Naming Battery (NNB); Thompson and Weintraub, 2014], with relatively mild impairments that did not differ significantly from the healthy controls. There also were no between-group differences on canonical and noncanonical sentence comprehension based on performance on the Sentence Comprehension Test from the NAVS, with both groups showing mild impairments, although scores for both sentence types were poorer for PPA-G than for PPA-L participants. However, the patients’ scores did not differ significantly from unimpaired adults. Semantic knowledge, as tested by the Pyramids and Palm Trees Test (PPT, picture version; Howard and Patterson, 1992) also was mildly impaired for both PPA groups relative to unimpaired participants (*ps* < 0.05 for both groups).

The major difference between the two patient groups pertained to sentence production. Mean scores on the Sentence Production Priming Test (SPPT) of the NAVS were poorer for PPA-G than for PPA-L participants (canonical sentences: PPA-G: *M* = 86.7, SD = 27.2; PPA-L: *M* = 96.7, SD = 6.5; noncanonical sentences: PPA-G: *M* = 62.0, SD = 30.3; PPA-G: *M* = 80.7, SD = 13.1), although these did not differ significantly. Sentence production for both groups was also impaired mildly for canonical sentences and more so for noncanonical sentences on the Northwestern Anagram Test (NAT; Thompson et al., 2012), with scores significantly different than unimpaired controls (*ps* < 0.05). Again, accuracy was poorer for the PPA-G group compared to the PPA-L group (canonical: PPA-G: *M* = 90.7, SD = 9.0; PPA-L: *M* = 96.0, SD = 4.7; noncanonical: PPA-G: *M* = 46.7, SD = 15.7; PPA-L: *M* = 80.7, SD = 13.1), though this difference only reached significance for the noncanonical sentences from the NAT (*p* < 0.05). In narrative language production (Cinderella story re-tell, analyzed using the Northwestern Narrative Language Analysis system; Thompson et al., 1995; Hsu and Thompson, 2018) both PPA groups showed significantly reduced speech rates (words per minute; Control: *M* = 134.7, SD = 16.3; PPA-G: *M* = 69.4, SD = 19.0; PPA-L: *M* = 81.6, SD = 25.8) and production of grammatical sentences (Control: *M* = 97.1%, SD = 4.3; PPA-G: *M* = 70.1%, SD = 23.9; PPA-L: *M* = 82.2, SD = 8.0) as compared to healthy controls, and did not differ from each other on these measures. Mean length of utterance (MLU, in words) did not differ significantly between the groups (Control: *M* = 11.3, SD = 3.8; PPA-G: *M* = 8.1, SD = 1.6; PPA-L: *M* = 8.9, SD = 1.9). Note that all scores showed quantitatively greater production deficits for the PPA-G compared to the PPA-L group.

### Materials

The stimuli consisted of 32 four-sentence stories, as in (1). In each story, the first sentence introduced two participants (e.g., *bride*, *groom*). The second sentence, a simple active, established an action (e.g., *tickling*) and the role that each participant played in the action (e.g., *bride* is the agent, *groom* is the theme). The third sentence introduced an unrelated participant (e.g., *clerk*). The fourth (target) sentence was created in two versions, with either a subject-relative clause (1a) or an object-relative clause (1b).

(1)

One day a bride and groom were walking in the mall.

The bride was feeling playful, so the bride tickled the groom.

A clerk was amused.

(a) Point to the one that [gap] was tickling the groom in the mall.
(b) Point to the one that the bride was tickling [gap] in the mall.

The participant nouns (e.g., *bride*, *groom*) were all 1–3 syllables. The agents and themes had dissimilar initial phonemes and were matched for length (agent: 1.66 syllables; theme: 1.66 syllables; unequal variance *t*-test: *t*_(61)_ = 0, *p* = 0.99) and natural log-transformed frequency from the 450 million word Corpus of Contemporary American English (COCA; agent participant: 9.27; theme participant: 9.01; unequal variance *t*-test: *t*_(62)_ = 0.64, *p* = 0.52). The nouns for the unrelated participant (e.g., *clerk*) met the same criteria and were not different in length (1.8 syllables; *ps* > 0.39) or natural log-transformed frequency (8.8; *ps* > 0.37) to the agent and theme nouns. The nouns were only used once each across the set of materials. To avoid bias due to the order of mention (Gernsbacher, 1989), the agent participant was introduced first in half of the stories; the theme participant was introduced first in the other half. The action verbs were all 1–2 syllable transitive verbs with regular past tense morphology. For the 32 stories, 28 different verbs were used; two were used twice and one was used three times.

Sixteen filler items also were constructed following the same format, except that there was only a single version of the target sentence, which referred to the unrelated participant from the third sentence (e.g., *cabbie*), as in (2).

(2)

One day, a woman and a student were visiting London together.

The student looked happy, so the woman photographed the student.

A cabbie drove them around.

Point to the one that was driving them around.

All stories were recorded by a female native English speaker at a normal speech rate (4–6 syllables per second). The rate of the subject-relative sentences (4.36 syllables per second) did not differ from the rate of the object-relative sentences (4.36 syllables per second; *t*_(60)_ = 0.08, *p* = 0.93).

**Figure 1:**
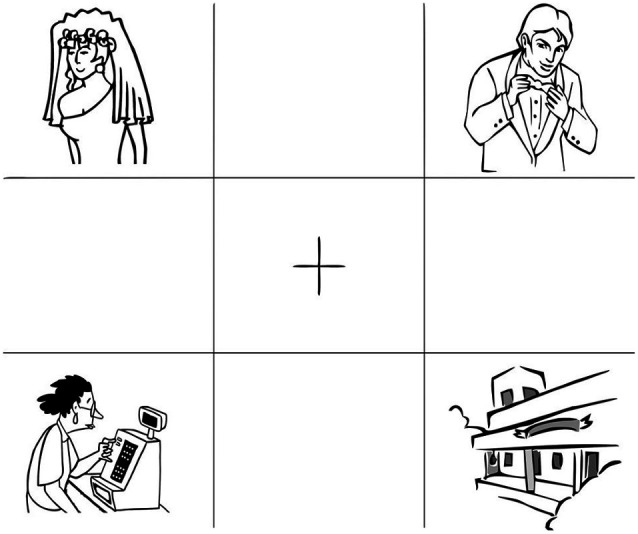
Sample visual array used in the visual world paradigm.

For each story, a visual array with pictures in the four corners and a central fixation cross was developed (Figure 1). The pictures were black and white line drawings and showed the agent and theme (e.g., *bride*, *groom*), the unrelated participant (e.g., *clerk*), and a scene or object mentioned in the first sentence (e.g., *mall*). The position of the correct picture and all other pictures was counterbalanced across visual arrays; all picture types (agent, theme, distractor, and location) occurred equally often in each of the four corner positions.

### Design

The stories were divided into two scripts of 48 items each. Conditions (i.e., sentence types) were counterbalanced across scripts and each included all 16 filler items pseudorandomly interspersed throughout. In each script, no more than two items of the same condition appeared in a row, and the correct picture was never in the same location for more than two consecutive stories. Each participant completed one or the other script in a single experiment.

### Procedure

Participants were seated in a dimly-lit room in front of a computer monitor, with their eyes level with the center of the computer screen and their chins placed in a chinrest, to reduce motion. An Applied Science Laboratories (ASL) 6000 remote eye-tracker was used to record the location of left eye fixations with a sampling rate of 60 Hz. Participants’ eyes were calibrated using a nine-point calibration screen at the beginning of the test session, with additional interim calibration checks every 10 trials.

Stimuli (stories and picture arrays) were presented by a computer using Superlab 4.0 (Cedrus). Participants were instructed to listen carefully to each story and click on the correct picture at the end of the story. A second computer was used for recording eye data and mouse click responses.

Participants used a mouse to click on the central fixation cross on the screen to begin each trial. After clicking, the cross remained on the screen for 1,000 ms, after which the array of pictures appeared. After another 1,000 ms, the auditory story began. The pictures remained on the screen throughout the presentation of the story, until the participant responded by clicking on one of the pictures. After the response (or after 10,000 ms elapsed with no response), the picture array was replaced with the central fixation cross. Participants clicked on the cross to begin the next trial at their own pace.

### Eye-Tracking Data Processing

We processed the data from the onset of the critical fourth sentence in each story. We used EYENAL (ASL) to first determine whether each sample point in the eye movement data (60 Hz sampling rate; 17 samples per second) was part of a fixation, defined as a gaze of at least 100 ms in duration within one degree of visual angle. Then we assigned each fixation to an area of interest in the visual array (i.e., each of the four corner pictures), created four variables corresponding to these regions of interest (fixation to agent, fixation to the theme, fixation to the distractor, fixation to location), and coded these variables for each sample point as 1 (fixation to this region) or 0 (fixation elsewhere). Consecutive data points are not necessarily independent but may exhibit dependencies reflecting constraints on how the eyes move. Given the relatively small number of participants and items, for each variable, we averaged successive data points for each item across 50 ms bins to filter out such dependencies (Barr, 2008). The data were time-locked to the offset of the word “that” at the start of the relative clause in each sentence condition, shifted by 200 ms to account for the time required to plan eye movements (Huettig and Altmann, 2005; Farris-Trimble and McMurray, 2013).

We computed the proportion of gazes to the target across sentences and participants by sentence type and group. Fixations from all sentences per condition, regardless of mouse click response accuracy, were included in eye data analyses.

## Analysis

### Response Accuracy

Mouse click responses to each item from each participant were coded as correct or incorrect with a binary variable. These data were analyzed separately for each sentence type with mixed-effects logistic regression models (SAS 9.4, proc glimmix) with crossed random effects of participant and item on the intercept and a fixed effect of group (healthy control, PPA-G, PPA-L). The models were fitted with an unstructured covariance matrix for each random effect. Results for the group effect are reported for the two regression coefficients, each specifying the difference between that group and the control group. Direct comparisons between the PPA-G and PPA-L groups were assessed with an estimate statement within the model. For each coefficient, we report the regression coefficient (B) of the contrast with its standard error, the *t*-test of the group difference, and the 95% confidence interval. Degrees of freedom were computed using the Satterthwaite approximation. The significance of all comparisons was assessed with *α* = 0.05. All *p*-values are reported two-tailed.

### Eye Movement Patterns

We analyzed the eye-movement data using linear growth curve models (SAS 9.4 proc glimmix) on the binned data (proportion of target fixations out of total fixations within each 50 ms bin per item) within specific regions of each sentence. For subject-relative clause sentences, we examined two regions, the first a post-gap region from the offset of the word “that” to the end of the direct object (e.g., “the groom”; post-gap; average duration across items: 1,334 ms), and the second a sentence-end region from the end of the direct object to the end of the sentence (average duration: 912 ms). For the object-relative clause sentences we examined three-sentence regions: the first encompassed the relative clause subject (subject; average duration: 583 ms), the next region included only the auxiliary and verb (verb; average duration: 742 ms), and the final region was from the gap to the end of the sentence (post-gap; average duration: 912 ms). Each region for each sentence type was analyzed in a separate model.

The growth curve models for each region within each sentence type were analyzed with fixed effects of group (control, PPA-G, PPA-L), time, and their interaction, as well as a random effect of participant on time, and random effects of participant and item on the intercept. The initial value of the time variable was reset to zero for each region, to facilitate the interpretation of the intercept. The models were fitted with a heterogeneous first-order autoregressive covariance matrix for each random effect, which allows for different variances at successive time points (−2 Residual Log Likelihood scores indicated that model fit was significantly improved for the heterogenous autoregressive matrix over a first-order equal-variance autoregressive matrix). Regression coefficients (B) were derived for each effect, and are each reported with their standard error, *t*-value with degrees of freedom, and the 95% confidence interval of the coefficient in the table of results for each sentence type. Degrees of freedom were computed using the Satterthwaite approximation. The significance of all comparisons was assessed with *α* = 0.05. We applied the Benjamini-Hochberg False Discovery Rate (FDR) correction (Benjamini and Hochberg, 1995) for multiple comparisons (with a FDR of 0.05) to all tests of slopes and intercepts within each sentence type and dependent variable. We, therefore, report *q*-values (two-tailed) instead of *p*-values.

We coded the factors included in the model to enable an interpretation of the slopes and intercepts for each participant group as follows. For the control group, the model intercept reflects the mean proportion of target gazes at the start of the region, and the coefficient of the main effect of Time reflects the rate of change (i.e., the slope) for the control group within the region. The significance of these effects (slope, intercept) was determined with *t*-tests against zero. The main effect of Group yielded two coefficients, one for the PPA-G group and another for the PPA-L group. Each of these coefficients reflected the difference for the patient group relative to the controls on the intercept. The Group by Time interaction also yielded two coefficients, each of which represents the difference in slope between a patient group and the control group: positive coefficients reflect a steeper slope than controls, negative coefficients a shallower slope. We refer to the coefficients from these factors as intercepts and slopes below. Direct *t*-test comparisons between the intercepts for PPA-G and PP-L groups (i.e., their coefficients from the Group effect) and their slopes (i.e., their coefficients from the Group by Time interaction) were assessed with estimate statements within each model.

### Timing of Gap Processing in Object-Relative Sentences

For this additional analysis, the data were smoothed using a five point moving average window; the first two and last two data points in the enlarged time window were removed. For the analysis, the time window was reset so that the gap occurred at time 0 and widened to include both the verb region (i.e., the region just before the gap) and the post-gap region. We computed the spline regression (SAS 9.4 proc nlin) for each participant with two parameters for the slopes of the two regression line segments in the pre-gap (i.e., verb) and post-gap regions (unlike the analyses reported above, the slopes here are computed relative to zero, not relative to the slope of the control group), and one parameter for the knot. The knot corresponds to the change point between the slope parameters, and the value of the knot parameter corresponds to the time (*x*-axis variable) at which there was a change in slope. The coefficients from linear spline models are therefore much easier to interpret than the coefficients from polynomial regression models (Mirman et al., 2008). For a similar approach with non-linear models, see Farris-Trimble and McMurray (2013). We extracted the individual knot parameter values for both the agrammatic and logopenic participants and used a *t*-test against 0 for each group to determine if that group’s knot was at a time that significantly differed from 0, consistent with delayed (or potentially early) gap processing.

## Results

### Response Accuracy

For subject-relative clauses (Table 2A), the PPA-G participants (79.4% correct) performed significantly more poorly than the PPA-L participants (90.0% correct; *p* = 0.02), and both groups were significantly worse than controls (97.4% correct; *p* < 0.0001 and *p* = 0.009 respectively). For object-relative clauses (Table 2B), both the PPA-G participants (70.6%) and the PPA-L participants (87.5%) were significantly less accurate than controls (98.8% correct; *p* = 0.0001 and *p* = 0.01 respectively), though for these sentences the PPA-G participants did not significantly differ from the PPA-L participants (*p* = 0.1).

**Table 2:** Mean percentage (and standard deviation) of correct and incorrect mouse click responses for subject-relative and object-relative sentences by participant group.

	CORRECT	INCORRECT		
	Agent	(Theme)	(Distractor)	(Other)
**A. Subject-relative**
Control (*n* = 15)	97.4% (3.3)	0.9% (2.4)	1.7% (2.9)	0 (0)
PPA-G (*n* = 10)	**79.4%**^1,2^ (12.5)	7.5% (7.1)	11.9% (6.9)	1.3% (2.6)
PPA-L (*n* = 10)	**90.0%**^2,3^ (10.7)	5.0% (6.5)	4.4% (5.1)	0.6% (2.0)
**B. Object-relative**
Control (*n* = 15)	98.8% (3.5)	0 (0)	0.8% (2.2)	0.4% (1.6)
PPA-G (*n* = 10)	**70.6%**^4,5^ (23.0)	17.5% (16.6)	8.8% (8.9)	3.1% (4.4)
PPA-L (*n* = 10)	**87.5%**^5,6^ (14.4)	8.1% (9.3)	2.5% (6.0)	1.9% (4.2)

*Incorrect responses are further categorized by the picture that was clicked (percentages across correct and incorrect responses may not add to 100% for each participant group due to rounding). ^1^PPA-G vs. control: *B* = −2.4 (0.51), *t*_(89)_ = 4.78, *p* < 0.0001; 95% CI: [−3.46, −1.42]. ^2^PPA-G vs. PPA-L: *B* = −1.0 (0.41), *t*_(27)_ = 2.43, *p* = 0.02; 95% CI: [−1.85, −0.16]. ^3^PPA-L vs. control: *B* = 1.4 (0.54), *t*_(108)_ = 2.68, *p* = 0.009; 95% CI: [0.38, 2.51]. ^4^PPA-G vs. control: *B* = −3.5 (0.85), *t*_(51)_ = 4.14, *p* = 0.0001; 95% CI: [−5.20, −1.80]. ^5^PPA-G vs. PPA-L: *B* = −1.1 (0.68), *t*_(17)_ = 1.75; *p* = 0.1, 95% CI: [−2.62, 0.24]. ^6^PPA-L vs. control: *B* = 2.3 (0.87), *t*_(55)_ = 2.64, *p* = 0.01; 95% CI: [0.87, 2.31]. Bolded values indicate significantly worse performance than controls (p < 0.05)*.

### Eye Movement Patterns

#### Subject-Relative Clauses

**Figure 2:**
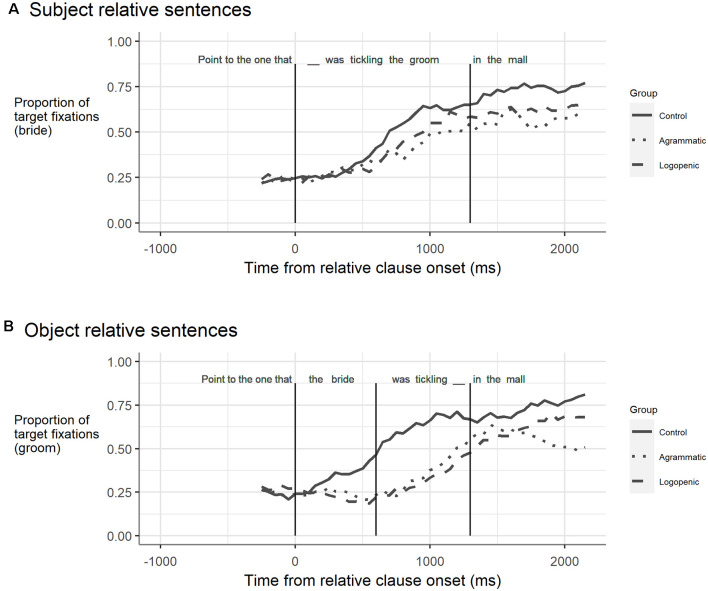
Fixations to the target over time for **(A)** subject-relative and **(B)** object-relative sentences for Control, Agrammatic, and Logopenic group-averaged data. Fixation data are shown starting 250 ms before the first analyzed time window. For subject-relative sentences, this was the verb and object (_—_*was tickling the groom*). Vertical lines correspond to the analyzed time windows and are at 0 and 1,300 ms. For object-relative sentences the first analyzed time window was at the subject (*the bride*); vertical lines demarking the time windows are at 0, 600, and 1,300 ms. Note that there were no pauses in audio at the gap positions (_—_).

At the onset of the post-gap region (i.e., starting at the gap), the control participants had begun to look at the target (Table 3, Figure 2A). Thus the intercept, which reflects the average proportion of target looks for the group, was significantly greater than zero: *B* = 0.184, *q* = 0.002. At this point, the two PPA groups did not differ from controls or each other (i.e., group differences on the intercept, which reflect group differences in the average target looks at the onset of the region: PPA-G: *q* = 0.92; PPA-L: *q* = 0.92; vs. each other, *q* = 0.97). Within this region, the control group significantly increased their looks to the target over time (slope: *B* = 0.0004, *q* = 0.001). The PPA-G and PPA-L groups also increased their proportion of looks to the target over time in this region, and their slopes did not differ from that of the controls (PPA-G: *q* = 0.14; PPA-L: *q* = 0.49), or between each other (PPA-G vs. PPA-L: *q = 0.74)*.

**Table 3:** Group differences for each analyzed sentence region from growth curve models of fixations over time to the filler (target) in subject-relative clause sentences^1^.

	Intercept	Intercept significance	Slope	Slope significance
**Subject-relative clause sentence: *point to the one that …***
**Post-gap region: _*was tickling the groom***
controls (vs. 0)	0.184* (0.05)	*t*_(41)_ = 3.88, *q* = 0.002	0.0004* (0.00006)	*t*_(32)_ = 7.64, *q* = 0.001
		95% CI: [0.09, 0.28]		95% CI: [0.0003, 0.0005]
PPA-G (deviation from controls)	−0.020 (0.07)	*t*_(33)_ = 0.28, *q* = 0.92	−0.0002 (0.00009)	*t*_(33)_ = 2.10, *q* = 0.14
		95% CI: [−0.12, 0.16]		95% CI: [−0.0004, −0.000006]
PPA-L (deviation from controls)	−0.017 (0.07)	*t*_(32)_ = 0.24, *q* = 0.92	−0.0001 (0.00009)	*t*_(32)_ = 1.21, *q* = 0.49
		95% CI: [−0.13, 0.16]		95% CI: [−0.0003, 0.00007]
PPA-G vs. PPA-L	0.03 (0.08)	*t*_(33)_ = 0.04, *q* = 0.97	0.0001 (0.00001)	*t*_(33)_ = 0.82, *q* = 0.74
		95% CI: [−0.16, 0.16]		95% CI: [−0.0001, 0.0003]
**Sentence-end region: *in the mall***	
controls (vs. 0)	0.714* (0.06)	*t*_(41)_ = 12.22, *q* = 0.001	0.00008 (0.00007)	*t*_(32)_ = 1.18, *q* = 0.49
		95% CI: [0.60, 0.83]		95% CI: [−0.00006, 0.0002]
PPA-G (deviation from controls)	−0.20 (0.09)	*t*_(32)_ = 2.34, *q* = 0.10	−0.00003 (0.0001)	*t*_(32)_ = 0.26, *q* = 0.92
		95% CI: [−0.38, −0.03]		95% CI: [−0.0002, 0.0002]
PPA-L (deviation from controls)	−0.14 (0.09)	*t*_(32)_ = 1.67, *q* = 0.28	+0.00001 (0.0001)	*t*_(32)_ = 0.13, *q* = 0.96
		95% CI: [−0.32, 0.03]		95% CI: [−0.0002, 0.0002]
PPA-G vs. PPA-L	0.06 (0.10)	*t*_(33)_ = 0.62, *q* = 0.87	0.00004 (0.0001)	*t*_(33)_ = 0.35, *q* = 0.92
		95% CI: [−0.14, 0.25]		95% CI: [−0.0002, 0.0003]

^1^Values are reported as the coefficient and standard error (in parentheses); significance is given by t-value, q-value, and 95% confidence interval. Significant coefficients are bolded and marked with *.

There were no group differences in the sentence-end region. At the start of the region, the controls had a high proportion of looks to the target (intercept: *B* = 0.714, *q* = 0.001). The intercept for the PPA-L group did not differ significantly from that of the control group (*q* = 0.28), neither did the intercept for the PPA-G group (*q* = 0.10), and the two PPA groups did not differ from each other (*q* = 0.87). Over time, the proportion of target looks did not change for the control participants (slope did not differ from zero: *B* = 0.00008, *q* = 0.49). The slope for the PPA-L group did not differ from the control slope (*q* = 0.96), nor did the slope for the PPA-G group (*q* = 0.92), and again, the two PPA groups did not differ from each other (*q* = 0.92).

#### Object-Relative Clauses

For the object-Relative clause sentences (Table 4, Figure 2B), all three groups had begun looking at the correct target at the onset of the relative clause subject: the control intercept was significantly higher than zero (*B* = 0.235, *q* = 0.0004), and the intercept for the PPA-G group did not differ significantly from that of the control group (*q* = 0.81), nor did the intercept for the PPA-L group (*q* = 0.75). The PPA-G and PPA-L groups did not differ at this point (group difference on intercept: *q* = 0.88). In this region, the control group significantly increased their proportion of looks to the target over time (slope: *B* = 0.00034, *q* = 0.0004). In contrast, the two PPA groups both decreased their proportion of target looks over time in this region. Group differences in the slope were significant relative to controls for the PPA-G group (*q* = 0.008) and the PPA-L group (*q* = 0.001). The slopes for the two PPA groups did not differ (*q* = 0.68).

**Table 4:** Group differences for each analyzed sentence region from growth curve models of fixations over time to the filler (target) in object-relative clause sentences.^1^.

	Intercept	Intercept significance	Slope	Slope significance
**Object-relative clause sentence: *Point to the one that …***
**Subject region: *the bride***
Controls (vs. 0)	0.235* (0.05)	*t*_(43)_ = 5.17, *q* = 0.0004	0.0003* (0.0001)	*t*_(29)_ = 4.65, *q* = 0.0004
		95% CI: [0.14, 0.33]		95% CI: [0.0002, 0.0005]
PPA-G (deviation from controls)	−0.019 (0.07)	*t*_(32)_ = 0.28, *q* = 0.81	−0.0004 (0.0001)	*t*_(31)_ = 3.25, *q* = 0.008
		95% CI: [−0.12, 0.15]		95% CI: [−0.0006, −0.0001]
PPA-L (deviation from controls)	−0.030 (0.07)	*t*_(33)_ = 0.45, *q* = 0.75	−0.0005 (0.0001)	*t*_(31)_ = 3.96, *q* = 0.001
		95% CI: [−0.11, 0.17]		95% CI: [−0.0007, −0.0002]
PPA-G vs. PPA-L	0.011 (0.07)	*t*_(33)_ = 0.15, *q* = 0.88	0.0001 (0.0001)	*t*_(33)_ = 0.67, *q* = 0.68
		95% CI: [−0.14, 0.16]		95% CI: [−0.0004, 0.0002]
**Verb region: *was tickling***	
Controls (vs. 0)	0.56* (0.05)	*t*_(45)_ = 10.83, *q* = 0.0004	0.0003* (0.0001)	*t*_(32)_ = 2.61, *q* = 0.03
		95% CI: [0.45, 0.66]		95% CI: [0.00006, 0.0005]
PPA-G (deviation from controls)	−0.36* (0.07)	*t*_(32)_ = 4.87, *q* = 0.0004	0.00024 (0.0002)	*t*_(33)_ = 1.53, *q* = 0.23
		95% CI: [−0.51, −0.21]		95% CI: [−0.00008, 0.0006]
PPA-L (deviation from controls)	−0.33* (0.07)	*t*_(32)_ = 4.45, *q* = 0.0004	0.00008 (0.0002)	*t*_(33)_ = 0.49, *q* = 0.75
		95% CI: [−0.48, −0.18]		95% CI: [−0.0002, 0.0004]
PPA-G vs. PPA-L	0.03 (0.08)	*t*_(33)_ = 0.39, *q* = 0.76	0.00016 (0.0002)	*t*_(34)_ = 0.94, *q* = 0.53
		95% CI: [−0.13, 0.20]		95% CI: [−0.0005, 0.0002]
**Post-gap region: _—_*in the mall***	
Controls (vs. 0)	0.67* (0.06)	*t*_(42)_ = 11.49, *q* = 0.0004	0.0002 (0.00008)	*t*_(32)_ = 2.06, *q* = 0.09
		95% CI: [0.55, 0.78]		95% CI: [0.000001, 0.0003]
PPA-G (deviation from controls)	−0.05 (0.08)	*t*_(31)_ = 0.61, *q* = 0.69	−0.0003 (0.0001)	*t*_(32)_ = 2.46, *q* = 0.02
		95% CI: [−0.22, 0.12]		95% CI: [−0.0006, −0.00005]
PPA-L (deviation from controls)	−0.18 (0.08)	*t*_(30)_ = 2.2, *q* = 0.07	+.0001 (0.0001)	*t*_(32)_ = 0.8, *q* = 0.60
		95% CI: [−0.35, −0.013]		95% CI: [−0.0002, 0.0004]
PPA-G vs. PPA-L	0.13 (0.09)	*t*_(31)_ = 1.43, *q* = 0.26	0.0004 (0.0001)	*t*_(33)_ = 2.97, *q* = 0.01
		95% CI: [−0.32, 0.056]		95% CI: [0.0001, 0.0007]

^1^Values are reported as the coefficient and standard error (in parentheses); significance is given by *t*-value, q-value, and 95% confidence interval. Significant coefficients are bolded and marked with *.

As a result of their increasing target looks during the relative clause subject, the control group began the verb region with a relatively high proportion of looks to the target (intercept: *B* = 0.56, *q* = 0.0004). In contrast, the proportion of target looks at the start of this region was significantly lower compared to controls for both the PPA-G group (*q* = 0.0004) and the PPA-L group (*q* = 0.0004); the two groups did not differ from each other (*q* = 0.76). In this region, the controls continued to significantly increase their proportion of target looks over time (slope: *B* = 0.0003, *q* = 0.03). Likewise, the proportion of target looks for both PPA groups increased over time similarly to the control group (slope difference vs. controls: PPA-G: *q* = 0.23; PPA-L: *q* = 0.75), and the two PPA groups did not differ from each other (slope difference across PPA groups: *q* = 0.53).

Immediately following the gap, the controls had a high proportion of looks to the target (intercept: *B* = 0.67, *q* = 0.0004). The proportion of target looks was lower than controls for both PPA groups, but the differences were not significant for either the PPA-G group (group difference on intercept: *q* = 0.69) or the PPA-L group (group difference on intercept: *q* = 0.07), and the intercepts for the two group did not differ from each other (*q* = 0.26). The proportion of target looks in this region did not significantly increase over time for the control group (slope: *B* = 0.0002, *q* = 0.09). A similar pattern was seen for the PPA-L group, whose slope did not differ from that of the controls (*q* = 0.60). However, the proportion of looks to the target decreased significantly over time for the PPA-G group relative to the controls (*q* = 0.02), and to the PPA-L group (*q* = 0.01).

#### Delayed or On-Time Gap Processing in Object-Relative Sentences?

We examined whether gap processing was delayed by investigating whether the change in regression slope from the verb region to the post-gap region in the object-relative sentences was co-incident with the gap or might have been delayed relative to the gap. The results indicate that the mean of the estimated change points was not significantly different than zero either for the PPA-G group (mean: 162 ms, SD: 892 ms; *t*_(9)_ = 1.56, *p* = 0.16) or the PPA-L group (mean: −104 ms, SD: 812 ms; *t*_(9)_ = 0.37 *p* = 0.72).

## Discussion

We used a visual world eye-tracking paradigm to examine the real-time processing and comprehension of complex sentences in individuals with PPA and healthy controls. Participants listened to short three-sentence stories about two participants (e.g., bride, groom), and then heard a final sentence asking them to point at one of the participants by clicking on the target picture in a visual array of four pictures. The final sentence was structured with either a subject-relative clause [*Point to the one (that_—_ tickled the groom) in the mall*] or an object-relative clause [*Point to the one (that the bride tickled_—_*) in the mall]. We measured response accuracy as well as eye-movements during the target sentences to examine how individuals with PPA process the structural gaps (indicated by “_—_” in the examples) in these relative clauses in real-time.

Concerning response accuracy, both patient groups showed poorer performance compared to healthy controls for both sentence types, as expected, with a more severe impairment for the PPA-G group, consistent with their performance on standard offline measures (i.e., comprehension and production accuracy at canonical sentences from the NAVS SPPT and the NAT, though this only reached significance for sentence production on the NAT; Table 1). This finding is also similar to that of *a prior* study showing (numerically) reduced accuracy of subject-relative clause comprehension in both PPA-G and PPA-L (Thompson et al., 2013). For the object-relative sentences, the PPA-G group also showed reduced response accuracy compared to the control participants, consistent with prior findings of noncanonical sentence comprehension deficits in PPA-G (Cooke et al., 2003; Wilson et al., 2010; Charles et al., 2013; Thompson et al., 2013; Kinno et al., 2017; but see Zimmerer et al., 2014). The PPA-L group also had impaired response accuracy relative to the control participants and were not statistically different from the PPA-G group.

Turning to the eye movement patterns, for the sentences with subject-relative clauses, the healthy control group steadily increased their rate of gazing towards the correct target immediately after the gap in the sentence, before it leveled off in the sentence-end region. The eye-movement patterns for the PPA-G and PPA-L groups were not statistically different from those of the control group, either in the post-gap region or the sentence-end region, particularly given the correction for multiple comparisons.

For the object-relative clause sentences, the healthy controls had a high (but non-increasing) rate of looks to the filler of the gap (e.g., *the groom*) in the post-gap region (e.g., *in the mall*). The PPA-L group was not different from the controls in this region, and also showed a high but steady rate of looks to the filler. In contrast, the PPA-G group showed a significantly decreasing rate of looks to the target, relative to both of the other groups. Additional spline regression analysis revealed that the slope changed and began to decrease at the gap position.

We argue that the thematic integration deficit account of agrammatic aphasia accounts for these results (Thompson and Choy, 2009). The finding that changes in the eye-gaze slopes began at the gap site is consistent with the prediction of intact filler-gap structure building. Also, the decreasing looks to the target in the post-gap region for the agrammatic comprehenders are consistent with the deficient interpretation of the filler’s thematic role at this point. That is, we take the change in processing that begins at the gap position as evidence that the gap itself was structured. If the gap had not been created, then it is not clear what kind of process could have led to the sudden change that we observed at that point. These results are consistent with prior findings from agrammatic stroke aphasia with this paradigm, which report normal gap-filling in the face of a reduced advantage for looks to the theme (relative to the agent) after the gap in stroke agrammatism for object-extracted wh-questions and cleft structures (Dickey et al., 2007; Dickey and Thompson, 2009). The correspondence in the patterns across populations is particularly clear when comparing the gazes over time to the theme. Just as for the current results for agrammatic PPA, the agrammatic stroke results from those studies also indicate a sharp downward turn for theme looks after the gap in object-relative sentences (Dickey and Thompson, 2009, Figure 2B).

**Figure 3:**
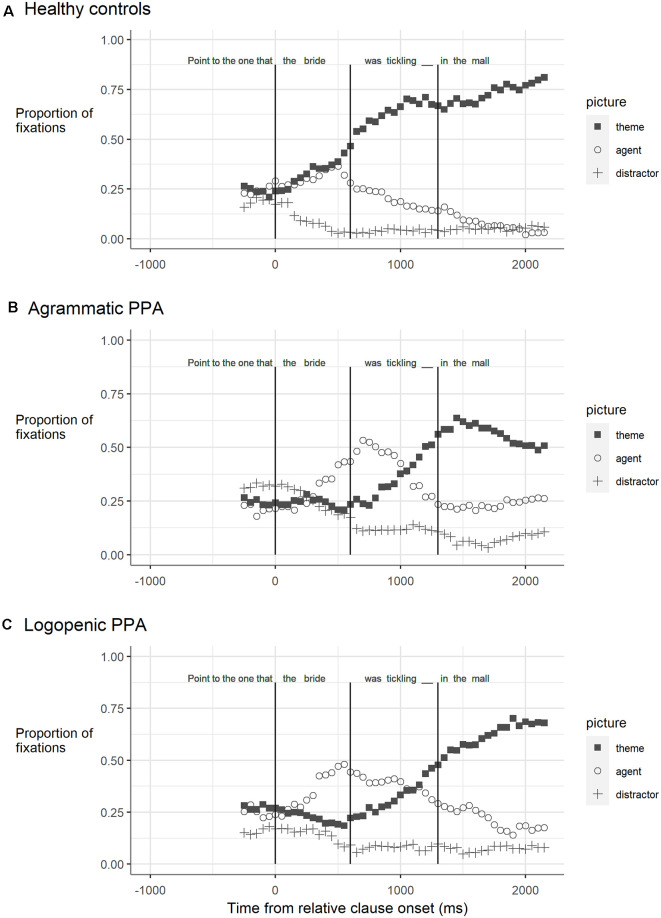
The proportion of fixations to the target (theme: *groom*), subject (agent: *bride*), and distractor pictures in object-relative sentences for **(A)** the healthy control participants; **(B)** the participants with agrammatic primary progressive aphasia (PPA); and **(C)** the participants with logopenic PPA. Vertical lines correspond to the analyzed time windows and are at 0, 600, and 1,300 ms.

A second aspect of the thematic integration deficit account that is supported by the current data is that predictions regarding the thematic role of the filler are impaired in agrammatic comprehension. A broader view of the eye-movement patterns before the gap in the object-relative sentences speak to this issue. During the relative clause subject (“*the bride*”), healthy control participants increased their gazes both to the filler (*groom*) and to the relative clause subject (*bride*, Figure 3A). This pattern is consistent with an agent-first strategy, given the potential for both animate participants (*groom, bride*) to be agents. Note that correctly predicting a theme role for the filler at this point would be unexpected—the sentence does not yet contain any information about which participant is linked to which thematic role. However, this pattern changed during the relative clause verb (e.g., “*was tickling*”), where looks to the agent (*bride*) decreased and looks to the theme (*groom*) continued to rise. This indicates that during the verb, healthy listeners correctly assign the agent role to the relative clause subject, leaving the correct prediction that, when encountered, the theme role would be assigned to the filler. This reflects a reanalysis process, whereby the initial assumption of an agent role is changed to an expected theme role for the filler. Confirmation of the filler’s theme role at the gap is also consistent with the finding of a steady rate of target-looks in the post-gap region.

In contrast, the participants with PPA showed a different pattern than the controls. While they are hearing the relative clause subject (e.g., “*the bride*”), looks to the corresponding picture (*bride*) increased, but looks to the correct filler (e.g., “*groom*”) decreased (Figures 3B,C). This indicates that, rather than using an agent-first strategy, correctly predicting that either participant could be the agent, their looks corresponded directly with the word heard at that point in the sentence. This pattern is consistent with a thematic prediction deficit. Notably, this pattern was found in the eye movements of both PPA-G and PPA-L participants, suggesting that this aspect of processing is not specific to agrammatism. This pattern also diverges from *a prior* finding in agrammatic stroke aphasia, where it was observed that looks to the theme and agent were similar (i.e., there was no preference) during the subject region of object wh-questions (Dickey et al., 2007; Dickey and Thompson, 2009).

However, in the subsequent relative clause verb region (e.g., “*was tickling*”), both PPA groups showed increased looks to the filler (*groom*) at a rate that was similar to that of the controls, reflecting thematic reanalysis in anticipation of a downstream object gap. However, after the gap, looks to the filler decreased in the agrammatic group, indicating erroneous thematic integration. Notably, the PPA-L group did not show this decrease. Rather they showed sustained looks to the filler, like the healthy controls. These findings indicate that whereas participants with PPA-G show impaired thematic prediction and integration, those with PPA-L evince difficulty only with thematic prediction.

One potential issue for this interpretation concerns a processing strategy that may be possible for our materials. That is, once the subject/agent has been identified, the preceding story and the meaning of the verb enable the theme to be correctly predicted as the target, even if the sentence structure is not processed beyond that point. Thus, looks to the target should continue to increase, even if a gap is not created. Such a prediction does seem to be made during the verb region—looks to the theme (the correct target) increase in this region for all three groups (although this prediction did not start as soon in PPA as in the controls, it was nevertheless apparent during the verb). However, we think this alternative account does not explain our results. First, evidence from many other studies indicates that healthy comprehenders reactivate the filler at the gap position in these structures (Swinney and Fodor, 1989; Love and Swinney, 1996; Nicol et al., 2006; Love, 2007; and references therein). This is interpreted as a reflex of automatic language processing. Thus, we expect that the healthy control participants are correctly structuring the sentences, even though a similar pattern of target looks could be expected for our materials if no structural gap were created. The participants with logopenic PPA did not differ from controls in this region, so we also expect that their processing is unimpaired concerning the gap. However, despite apparently beginning to correctly predict the target during the verb, the participants with agrammatic PPA (who are predicted to have trouble structuring the gap on some views) began to look away from the correct target following the gap position—indicating that some element within the sentence disrupted their processing. This alternative account, which predicts continued looks to the target in the absence of a gap, therefore does not predict the pattern that we observed.

Instead, we argue that the present finding indicates that people with PPA do show evidence of gap-filling in object-relative sentences and that they do not show an agent-first strategy during online sentence comprehension. Thus our evidence does not support the predictions of the trace-deletion hypothesis (Grodzinsky, 1986, 1995). Likewise, the finding that gap-filling was on-time is contrary to the predictions of the slow-syntax and slow-rise hypotheses (Burkhardt et al., 2008; Love et al., 2008). In the current study, the speech rate of the auditory sentences was within the normal range, but participants show evidence of gap-filling at the gap site, as do healthy listeners, suggesting that this factor does not underlie sentence comprehension difficulty.

In conclusion, the eye movement patterns found in the present study suggest that the real-time processing of complex syntactic structures is impaired in agrammatic PPA, consistent with the hypothesis of deficient thematic integration. This includes deficits in both thematic prediction and post-verbal thematic integration, in the face of normal-like gap-filling processes during object-relative clause computation. These abnormal processing patterns help to explain the source of comprehension failure in PPA-G patients, and are in keeping with deficit patterns seen in stroke-induced agrammatic aphasia during the processing of syntactically complex structures. The novel finding that patients with PPA-L also show thematic prediction impairments may, at least in part, explain their difficulty in comprehending complex grammatical constructions. The clinical profiles of agrammatic and logopenic PPA, therefore, appear to overlap in some, though not all, aspects of syntactic processing. The implications of this finding for diagnosis and treatment of these different subtypes of PPA are left for future investigations.

## Data Availability Statement

The raw data supporting the conclusions of this article will be made available by the authors, without undue reservation.

### Ethics Statement

The studies involving human participants were reviewed and approved by The IRB of Northwestern University. The patients/participants provided their written informed consent to participate in this study.

## Author Contributions

All authors contributed to the article and approved the submitted version.

## Conflict of Interest

The authors declare that the research was conducted in the absence of any commercial or financial relationships that could be construed as a potential conflict of interest.
